# Imported Monkeypox, Singapore

**DOI:** 10.3201/eid2608.191387

**Published:** 2020-08

**Authors:** Sarah Ee Fang Yong, Oon Tek Ng, Zheng Jie Marc Ho, Tze Minn Mak, Kalisvar Marimuthu, Shawn Vasoo, Tsin Wen Yeo, Yi Kai Ng, Lin Cui, Zannatul Ferdous, Po Ying Chia, Bryan Jun Wei Aw, Charmaine Malenab Manauis, Constance Khia Ki Low, Guanhao Chan, Xinyi Peh, Poh Lian Lim, Li Ping Angela Chow, Monica Chan, Vernon Jian Ming Lee, Raymond Tzer Pin Lin, Mok Kwee Derrick Heng, Yee Sin Leo

**Affiliations:** Ministry of Health Singapore, Singapore (S.E.F. Yong, Z.J.M. Ho, C.K.K. Low, G. Chan, X. Peh, V.J.M. Lee, M.K.D. Heng);; National Centre for Infectious Diseases, Singapore (O.T. Ng, T.M. Mak, K. Marimuthu, S. Vasoo, T.W. Yeo, Y.K. Ng, L. Cui, Z. Ferdous, P.Y. Chia, B.J.W. Aw, C.M. Manauis, P.L. Lim, L.P.A. Chow, M. Chan, R.T.P. Lin, Y.S. Leo);; National Public Health Laboratory, Singapore (T.M. Mak, Y.K. Ng, L. Cui, R.T.P. Lin);; Tan Tock Seng Hospital, Singapore (O.T. Ng, K. Marimuthu, S. Vasoo, T.W. Yeo, Z. Ferdous, P.Y. Chia, B.J.W. Aw, C.M. Manauis, P.L. Lim, L.P.A. Chow, Y.S. Leo);; National University Health System, Singapore (S.E.F. Yong);; Nanyang Technological University, Singapore (O.T. Ng, P.Y. Chia, P.L. Lim, Y.S. Leo);; National University of Singapore, Singapore (K. Marimuthu, P.L. Lim, Y.S. Leo)

**Keywords:** Monkeypox, zoonoses, viruses, monkeypox virus, orthopoxvirus, disease outbreaks, imported infectious diseases, communicable disease control, Singapore, Nigeria

## Abstract

In May 2019, we investigated monkeypox in a traveler from Nigeria to Singapore. The public health response included rapid identification of contacts, use of quarantine, and postexposure smallpox vaccination. No secondary cases were identified. Countries should develop surveillance systems to detect emerging infectious diseases globally.

Monkeypox is a zoonosis endemic to West and Central Africa; human cases were first reported in 1970 (*1*). An outbreak ongoing in Nigeria since 2017 is the largest documented (*2*). Exported cases in the United Kingdom and Israel were reported from travelers infected in Nigeria in 2018 (*3*,*4*). An earlier outbreak of human cases in the United States in 2003 was linked to contact with prairie dogs infected by rodents from Ghana (*5*).

Singapore is a globally connected city-state in Southeast Asia, placing it at risk for importation of emerging infectious diseases. For this reason, the Singapore Ministry of Health informed frontline medical practitioners in 2018 of the risk for monkeypox. Seven months later, a case of travel-associated monkeypox was diagnosed in Singapore. We present case details and public health management for this case, together with lessons learned and implications for control.

## The Case

On May 8, 2019, monkeypox was laboratory-confirmed in a 38-year-old man from Nigeria who had traveled to Singapore. The man resided in Delta State, Nigeria, but had attended a wedding in Ebonyi State during April 21–23, where he reported ingestion of barbecued bushmeat that might have been contaminated. He did not handle raw meat and had no exposure to wild animals or their products. He held an administrative job and reported no contact with rodents or with persons with pox-like illnesses.

The man arrived in Singapore on April 28 and attended a business workshop on April 29–30. Fever, chills, and myalgia developed on April 30, and a vesicular rash on his face developed on May 1 and progressed cephalocaudally. After symptoms developed, he remained in his hotel room most of the time. He sought medical attention on May 7; an ambulance transported him to the Tan Tock Seng Hospital emergency department, and he was transferred to the adjacent National Centre for Infectious Diseases. The same day, the treating physician notified the Ministry of Health about the suspected monkeypox case.

**Figure 1:**
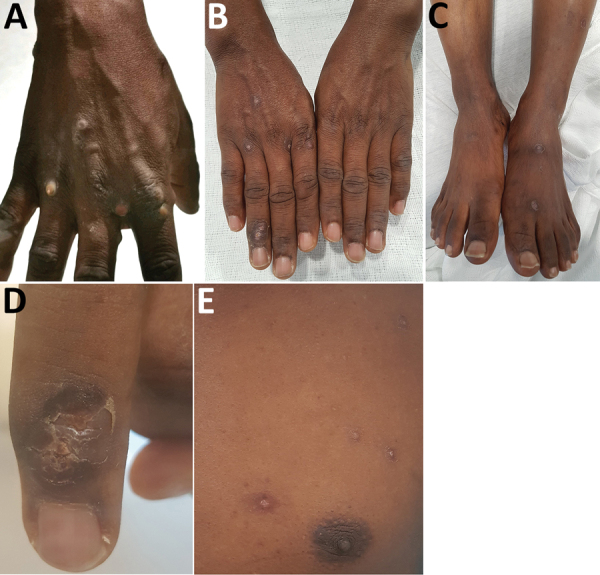
Dermatologic features of monkeypox in a 38-year-old man, Singapore, 2019. A) Pustular lesions on the hand at the start of hospitalization. B, C) Resolving lesions with shedding of scabs of the hands (B) and feet (C) toward end of hospitalization (day 17). D, E) Crusting of right fourth finger lesion (D) and lesions at varying stages (vesicles and scabbing) on the left chest (E) on day 15 of hospitalization.

At examination in the emergency department, the patient had a fever of 37.7°C. He had multiple pustular lesions of varying stages over his face, trunk, and limbs (Figure 1, panel A), including palms and soles, penile shaft, and glans penis. There was no oral involvement. Cervical and inguinal lymphadenopathy were present.

**Figure 2:**
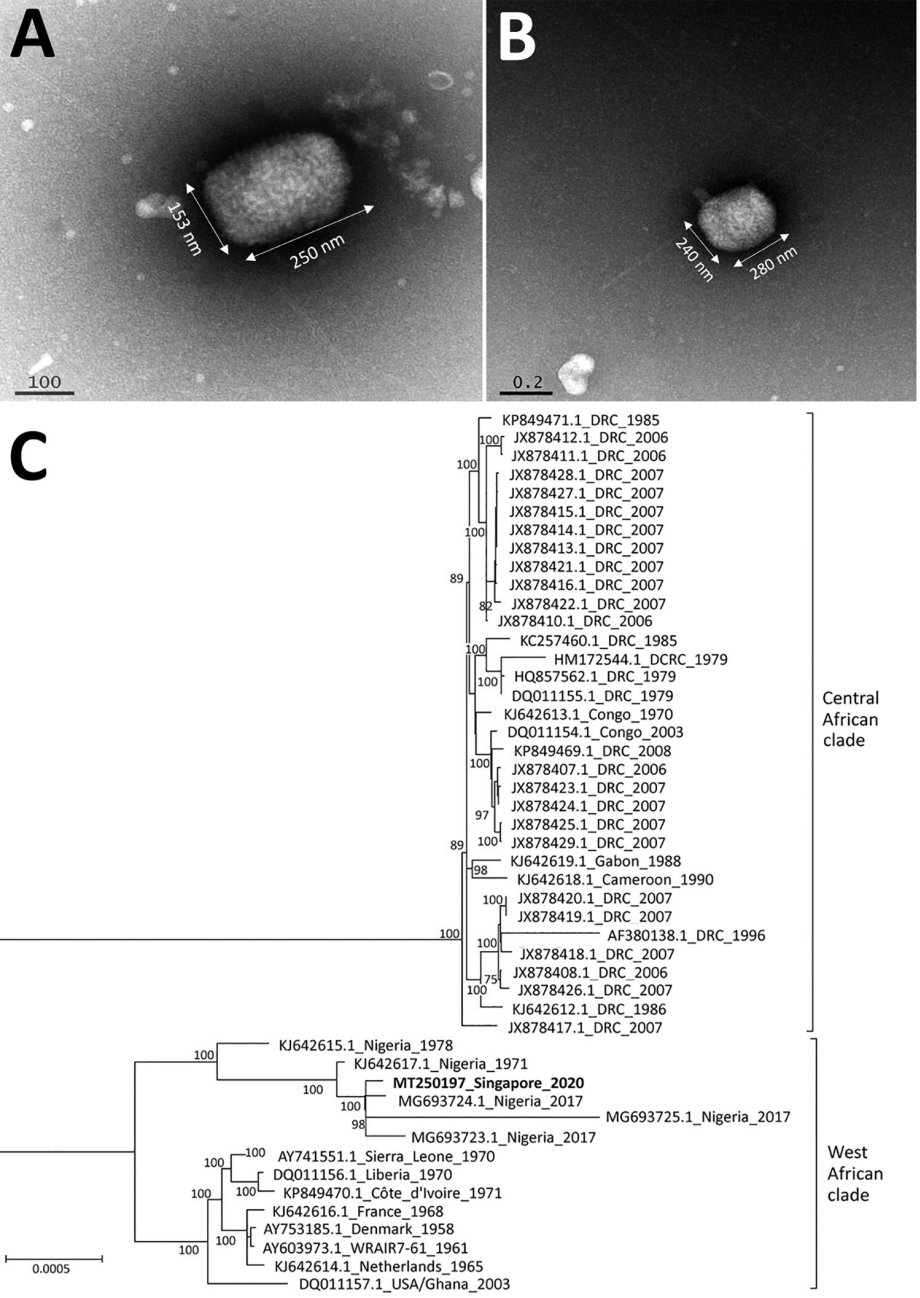
Transmission electron microscopy and maximum-likelihood phylogenetic tree of monkeypox virus in 38-year-old man, Singapore, 2019. A, B) Multiple brick-shaped particles, ranging from 230–290 nm by 130–240 nm, were observed from vesicle fluid under transmission electron microscopy. Tubular structures were observed with phosphotungstic acid stain (A), and a central ring-like depression was observed with gadolinium acetate stain (B). C) Phylogeny of monkeypox sequences, with the patient’s monkeypox strain in bold. All strains are identified by GenBank accession number, location, and year. The evolutionary relationships between monkeypox strains was determined based on 184,338 bases within the central core region of the monkeypox genome. The maximum-likelihood tree was created using RAxML (*10*) with γ-distributed rate differences and 1,000 bootstrap validation. Only bootstrap values >70% are displayed on the internal branches. Central African and West African clades are indicated. Scale bar indicates genetic distance between sequences.

In view of his travel history and symptoms, differential diagnoses considered included monkeypox and other poxvirus infections. Whole blood and swab specimens of the lesions and vesicle fluid were sent to the National Public Health Laboratory, where tests for orthopoxvirus were positive at 2 hours and monkeypox virus at 6 hours after specimen receipt. Serum and swab specimens tested positive for orthopoxvirus but negative for variola virus on the BioFire FilmArray Biothreat Panel version 2.5 (https://www.biofiredx.com). We confirmed orthopoxvirus from swab specimen using a panorthopoxvirus PCR targeting E9L (DNA polymerase) (*6*) and by direct visualization of virus particles using transmission electron microscopy, which showed features characteristic of orthopoxviruses (Figure 2, panels A, B) (*7*).

Detailed molecular analysis using PCR for monkeypox was positive for 2 monkeypox genes (B6R and B7R) (*8*). We further confirmed monkeypox by next-generation sequencing using the Illumina MiSeq platform (https://www.illumina.com). Sequencing read analysis showed alignment with monkeypox virus. The assembled sequence covered 98% of the closest genome reference on GenBank (accession no. KJ642617.1, a strain from Nigeria), with 99.96% identity. We aligned the consensus sequence with selected representative archived sequences with multiple alignment using Fast Fourier Transform (*9*) and created a maximum-likelihood tree using RAxML (*10*) with γ-distributed rate differences and 1,000 bootstrap validation. The virus belonged to the West African clade and clustered with strains from Nigeria with 100% bootstrap support (Figure 2, panel C).

The patient was isolated in a negative-pressure room and remained well throughout admission. He was examined daily for new pustules and evolution of scabs (Figure 1, panels B–E). By May 24, all scabs had shed, and he was de-isolated and discharged.

Within 24 hours after notification of the suspected monkeypox case, the Ministry of Health contact tracing team established the patient’s activities, identified contacts, and determined risk categories. These activities were performed through interviews with the patient and with his contacts. Close contacts were defined as persons who were within 2 meters of the patient for >30 minutes or had physical contact with him or had physical contact with surfaces or materials contaminated by secretions from him from April 30 on (*11*).

We identified 23 close contacts (19 workshop attendees and 4 hotel staff) and 8 lower risk contacts. Quarantine orders were issued to 22 close contacts on May 9; they were required to remain at home or at a government quarantine facility for the duration of the remaining incubation period (21 days). They were not allowed to come into physical contact with others on the same premises and were monitored for fever and rash >3 times each day through video calls. One close contact, who was well, had left Singapore for Nigeria on May 5; we provided details to the Nigerian International Health Regulations National Focal Point.

Close contacts were offered smallpox vaccination (ACAM2000; Sanofi Pasteur Biologics Co, https://www.sanofi.com) as postexposure prophylaxis. Of the 22 close contacts, 14 received the vaccination, 2 had contraindications, and 6 declined. All vaccinated persons had a scab or ulcer at day 6–8 of review. Side effects included slight fever and mild swelling at the vaccination site; no serious adverse events were reported. Lower risk contacts were placed on phone surveillance twice a day for the remaining incubation period.

Because of the early suspicion of an infectious disease, all healthcare workers who interacted with the patient used personal protective equipment. Ambulance paramedics and Tan Tock Seng Hospital emergency staff had worn N95 masks and disposable gowns and gloves. At the National Centre for Infectious Disease, healthcare workers donned full personal protective equipment (N95 mask, eye protection, disposable headgear, gloves, and sterile disposable gowns). Thus, no healthcare workers were quarantined or removed from work, but they were monitored for symptoms as an added precaution.

We followed up all contacts for 21 days after exposure. Monkeypox did not develop in any contacts, and we found no evidence of secondary transmission in Singapore.

## Conclusions

The patient’s clinical manifestations of a vesiculopustular rash and uncomplicated illness was similar to monkeypox cases in the United Kingdom and Israel, which were also linked to travel from Nigeria (*3*,*4*,*12*). All exported cases were of the West African clade, which is thought to be milder and less transmissible than the Congo Basin clade (*13*). Nevertheless, human-to-human transmission had been also demonstrated in Nigeria and the United Kingdom.

Singapore’s experience with monkeypox highlights the critical role frontline clinicians play in surveillance of emerging infectious diseases. This situation was similar to the 2016 Zika outbreak in Singapore, when a general practitioner contacted the Ministry of Health about an unusual increase in persons with fever, rash, and joint pains (*14*). In both instances, the Ministry of Health had informed physicians about the evolving global situation. Public health agencies should prioritize regular communication with healthcare workers as integral to preparedness for emerging infectious diseases.

Unlike previous monkeypox outbreaks, the outbreak in Nigeria affected predominantly urban dwellers and resulted in exported cases to geographically disparate countries (*1*). Increasing urbanization and better connectivity can lead to the emergence and spread of infections to new areas (*15*). Our experience with monkeypox highlights the importance of countries investing in preparedness, including maintaining surveillance systems suited to detecting the emergence of infectious diseases globally.
